# Exploring the role of EBV in multiple sclerosis pathogenesis through EBV interactome

**DOI:** 10.3389/fimmu.2025.1557483

**Published:** 2025-04-02

**Authors:** Chiara Ballerini, Roberta Amoriello, Olfa Maghrebi, Gianmarco Bellucci, Ilaria Addazio, Matteo Betti, Maria Grazia Aprea, Camilla Masciulli, Arianna Caporali, Valeria Penati, Clara Ballerini, Ermelinda De Meo, Emilio Portaccio, Marco Salvetti, Maria Pia Amato

**Affiliations:** ^1^ Department of Neuroscience, Psychology, Drug Research and Child Health (NEUROFARBA), University of Florence, Florence, Italy; ^2^ Department of Experimental and Clinical Medicine, University of Florence, Florence, Italy; ^3^ Department of Neurosciences, Mental Health and Sensory Organs, Sapienza University of Rome, Rome, Italy; ^4^ Neuromed, IRCCS Istituto Neurologico Mediterraneo (INM), Pozzilli, Italy; ^5^ Istituti di Ricovero e Cura a Carattere Scientifico (IRCCS) Fondazione Don Carlo Gnocchi, University of Florence, Florence, Italy

**Keywords:** interactome, MS pathogenesis, EBV, CMV, immune cells

## Abstract

**Background:**

Epstein-Barr virus (EBV) is a known risk factor for multiple sclerosis (MS), even though the underlying molecular mechanisms are unclear and engage multiple immune pathways. Furthermore, the ultimate role of EBV in MS pathogenesis is still elusive. In contrast, Cytomegalovirus (CMV) has been identified as a protective factor for MS.

**Objectives:**

This study aims to identify MS-associated genes that overlap with EBV interactome and to examine their expression in immune and glial cell subtypes.

**Methods:**

We used P-HIPSTer, GWAS, and the Human Protein Atlas (HPA) to derive data on the EBV interactome, MS-associated genes and single-cell gene expression in immune and glial cells. The geneOverlap and dplyr R packages identified overlapping genes. A similar analysis was done for CMV and Adenovirus as negative control. Metascape and GTEx analyzed biological pathways and brain-level gene expression; transcriptomic analysis was performed on glial cells and peripheral blood in MS and controls. All the analyses performed in this study were generated using publicly available data sets.

**Results:**

We identified a “core” group of 21 genes shared across EBV interactome, MS genes, and immune and glial cells (p<0.001). Pathway analysis revealed expected associations, such as immune system activation, and unforeseen results, like the prolactin signaling pathway. BCL2 in astrocytes, MINK1 in microglia were significantly upregulated while AHI1 was downregulated in MS compared to controls.

**Conclusions:**

Our findings offer novel insights into EBV and CMV interaction with immune and glial cells in MS, that may shed light on mechanisms involved in disease pathophysiology.

## 1 Introduction

Multiple sclerosis (MS) is an immune-mediated disease that affects the central nervous system (CNS). A complete comprehension of MS etiology is still missing (1). A pivotal aspect in this field is the association between the Epstein-Barr virus (EBV) and the disease, as EBV, a DNA virus belonging to the herpes virus family, is a recognized risk factor for MS development (2). Recent epidemiological studies have confirmed that EBV infection, especially if symptomatic (infectious mononucleosis), raises the risk of developing MS in susceptible individuals. The study by Bjornevik et al. (3) not only confirmed the association between EBV and the disease but also highlighted a causal relationship, demonstrating that the viral infection precedes the onset of the disease. Moreover, it revealed that the levels of Neurofilament Light chain (NfL), a nonspecific marker of damage in the CNS, were elevated in individuals who tested positive for EBV infection long before the disease onset. Such an increase was not observed in those EBV-positive individuals who did not develop MS later in life (3).

Different studies have identified the presence of EBV in the MS brain as well as in MS-associated meningeal follicles (4). These observations, initially controversial (5), have been recently confirmed (6). The possible presence of EBV in microglial cells and the virus role in inducing pro-inflammatory cellular phenotypes in glial cells are also observations of potential interest (7).

The mechanisms through which EBV may contribute to the onset and progression of inflammatory processes are not fully understood. To shed light on them it would be crucial to determine the role of immune response dysregulation and complex virus-host interaction. Molecular mimicry mechanisms have been identified as potential triggers of autoimmune responses in diseases as occurring in other immunological diseases (e.g. Guillain Barré Syndrome with *Campylobacter jejuni*) (8). Building on these premises, Vietzen and colleagues endeavored to discern the factors contributing to disease development and those exerting a protective role (9–11). For instance, while EBV represents a risk factor, Cytomegalovirus (CMV) appears to act as a protective factor (12). Thus, the role of CMV in MS etiology and disease modulation remains controversial (13). While some studies support a protective role for CMV in MS (14), others highlight its potential detrimental impact (15).

Overall, the infection by these pathogens seems to modulate response mechanisms in either a pro-inflammatory or anti-inflammatory direction, influencing the intercellular cross-talk within the immune system (9). Investigating how the virus-host interaction mechanisms may, through yet-to-be-explored pathways, be responsible for triggering the disease is of great interest. Equally stimulating is the hypothesis that EBV may generate aberrant responses, directly or indirectly, in immune cells and glial cells.

The emerging field of “network medicine” utilizes tools and concepts from network theory to study interactomes, that is networks encompassing various types of interactions, including protein-protein interactions occurring within a cell. Computational analyses have demonstrated how viruses efficiently maximize their effects by targeting multifunctional and highly connected host proteins.

In diseases that are potentially triggered by viruses, the construction of virus-host interactomes can therefore be helpful to better understand the pathogenetic mechanisms and identify potential pharmacological targets (16, 17). In the present study, we investigated the interaction of EBV with MS-associated genes and with immune and glial cells known as relevant in MS pathogenesis. While EBV is associated with various autoimmune diseases, this study focuses on its role in MS, a CNS-specific disease driven by genetic susceptibility, environmental triggers, and immune dysregulation. MS involves unique processes like immune activation, neuroinflammation, and viral interactions, particularly with EBV. Unlike CMV, whose association with MS remains controversial, EBV is strongly linked to MS development, with evidence of abnormal B-cell activation and persistence of EBV-infected cells in the CNS.

Given MS’s complexity, network-based bioinformatics is uniquely suited to explore its pathogenesis. Unlike traditional gene-focused analyses, this approach identifies key regulatory hubs, pathway disruptions, and broader molecular interactions, providing deeper insights into EBV’s role in MS. To ensure comprehensive and unbiased analysis, we investigated both EBV and CMV interactions with MS-associated genes and immune/glial cells relevant to MS pathogenesis.

## 2 Methods

### 2.1 Experimental workflow

**Figure 1:**
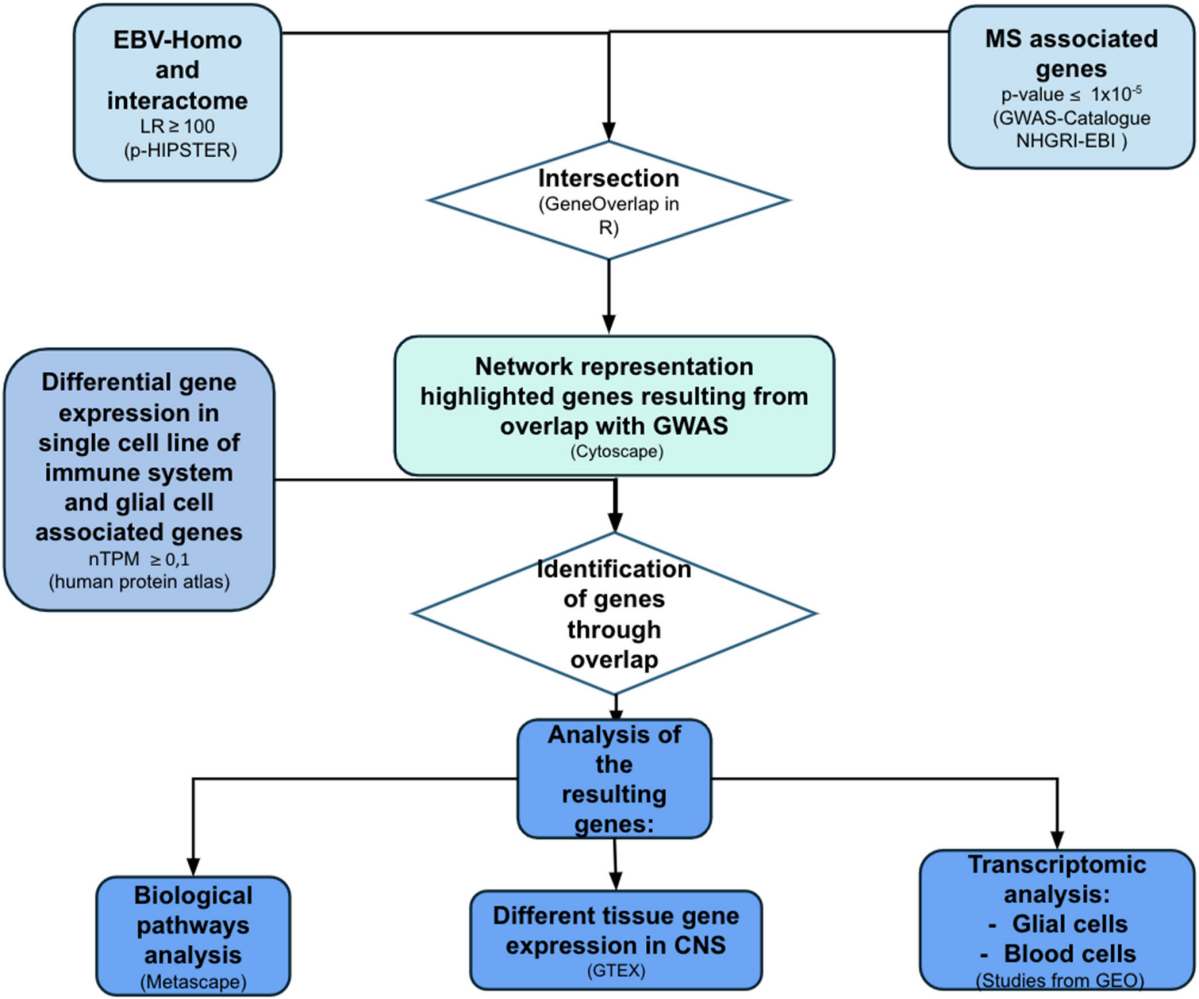
Flow-chart representing methods of creation and analysis of EBV-human interactome. The same process was applied to CMV analysis. LR, likelihood ratio; P-HIPSTer, Pathogen Host Interactome Prediction using STructurE similarity; GWAS, genome-wide association studies; nTPM, normalized Transcripts Per Million; GTEX, Genotype-Tissue Expression (made with pptx).

A structured data integration process was employed to allow the identification of the genes associated with increased MS susceptibility in the EBV-human interactome (**Figure 1**). The different degree of expression of these genes in blood and glial cells was therefore explored. The biological pathways in which these genes of the two viruses are involved, and the different patterns of expression at the CNS level, were investigated. Possible alterations of these genes in subjects with MS compared to controls were assessed through transcriptomic studies.

### 2.2 Virus-host interaction protein networks

The virus-host interaction through protein-protein interaction (PPI) data was analyzed from online databases (18–20). EBV (human gamma herpesvirus 4), CMV, and Adenovirus 7 data were downloaded on June 7^th^ 2023 from the Pathogen Host Interactome Prediction using STructurE similaRity (P-HIPSTer; available on http://phipster.org/). As negative control we used Adenovirus 7, a double-stranded DNA virus that causes a variety of diseases, including respiratory disorders, gastroenteritis, conjunctivitis, and hemorrhagic cystitis (21). The minimum likelihood ratio (LR) for the interaction between viral and human cell proteins was set as ≥ 100.

Network visualization was performed using Cytoscape (version 3.10.1), an open-source software platform for visualizing complex networks (22). Cytoscape was furtherly used to identify key nodes through the Betweenness centrality [CentiSCape APP version 2.2, (23)], which assesses the significance of a node based on the number of shortest paths that pass through it.

Given the major interest in the EBV role in MS, the following analyses will primarily focus on this virus, while also exploring some aspects involving CMV. On the other hand, Adenovirus 7 will not be discussed, as it did not show statistically significant overlap with EBV and MS-associated genes and serves as a negative control to MS comparison.

### 2.3 Overlap with GWAS

The mapped single-nucleotide polymorphisms (SNPs) genes associated with MS susceptibility (p-value ≤ 1x10^-5^) were extracted from the NHGRI-EBI Catalog of human genome-wide association studies (GWAS) by “Multiple Sclerosis” as keyword (24). The downloaded database obtained a total of 576 associated mapped genes for MS. All the detected MS-associated genes were maintained to ensure inclusivity.

Identification of genetic associations with the disease is still underway (25); in other recent studies, GWAS has been selected as a starting point to then investigate and identify genes of interest (26). An overlap analysis was performed between the EBV interactome and susceptibility genes for MS obtained from GWAS studies, thus leveraging an “MS-specific” EBV interactome. This analysis was conducted using the GeneOverlap 1.38.0’ package in R (Version 2023.06.1 + 524). The same analysis was run for CMV and Adenovirus 7.

### 2.4 Gene expression in cell lines

From the Human Protein Atlas [Human Protein Atlas, proteinatlas.org, version 23.0 (27)], the gene expression of individual cell lines of interest for MS pathogenesis was selected: B and T lymphocytes, macrophages, monocytes, dendritic cells, granulocytes, microglia, astrocytes, oligodendrocytes, and oligodendrocyte precursors. Genes with nTPM ≥ 0.1 were selected. The overlap analysis between the “MS-specific” EBV interactome (obtained as described above) and the selected gene-cell lists was conducted by the GeneOverlap 1.38.0’ package in R (Version 2023.06.1 + 524). Subsequently, we sought common genes across different cells by an overlap analysis using the dplyr version 1.1.4’ package in). The same analysis was performed for CMV, identifying a gene pool for all different cell subtypes. The comparison between the two viruses was depicted by Venn diagrams by the ggvenn R package (n 0.1.10).

### 2.5 Pathway analysis, tissue gene expression and transcriptomics

The biological pathways of the identified genes were investigated by Metascape (28). To compare different pathways for CMV and EBV the enrichplot R package was used, which works with results from clusterProfiler and supports visualization of enrichment results in a network structure based on the respective pathway enrichment results from Reactome, GO, and WikiPathway of CMV and EBV genes associated with MS and intersected with single cell immune genes. The analysis of tissue gene expression was performed by the Genotype-Tissue Expression (GTEx) Portal (29). We selected tissue from different brain regions, namely cerebellum, cerebellar hemisphere, anterior cingulate cortex, frontal cortex, brain cortex, spinal cord, hippocampus, amygdala, substantia nigra, hypothalamus, putamen, nucleus accumbens and caudate. We also included blood cells and EBV-infected B lymphocytes; these were used as negative and positive controls, respectively.

Finally, transcriptomic studies were analyzed to assess whether the examined genes for EBV were differentially expressed in MS patients compared to controls. In particular, we selected the series GSE111972 from the NCBI Gene Expression Omnibus Database, including transcriptional profiling of *post-mortem* brain sample, specifically from 31 human microglia samples belonging to 10 MS donors, including 5 gray matter and 10 white matter samples, and from 11 control donors, including 5 gray matter and 11 white matter samples (30). For astrocytes, we selected the series GSE83670 from NCBI Gene Expression Omnibus Database. The samples were derived from 10 secondary-progressive MS (SPMS) and 9 controls. Specifically, the expression profiling of astrocytes was derived from MS Normal Appearing White Matter (NAWM) and from Neurologically normal control white matter (WM) (31). For peripheral blood cell lines, we performed the analysis on downloaded published data from David Schafflick et al., 2020, deposited on GEO with the code GSE138266 (32). For oligodendrocytes, we selected the series GSE118257 on GEO (33). Transcriptomic datasets were reanalysed through GEOR tool and R limma package (version 3.54.2).

For all these analyses, we set a threshold of ±0.5 for log_2_FoldChange. P-values were adjusted for multiple comparisons using Benjamini & Hochberg false discovery rate (FDR). Statistical significance was considered when p-value < 0.05.

## 3 Results

### 3.1 EBV and CMV interactome overlap with GWAS

Our analysis focused on EBV given its strength of association with MS, but also explored CMV, another herpesvirus, which has implicated in the regulation mechanisms of the immune system and the physiopathology of the disease (34).

**Figure 2:**
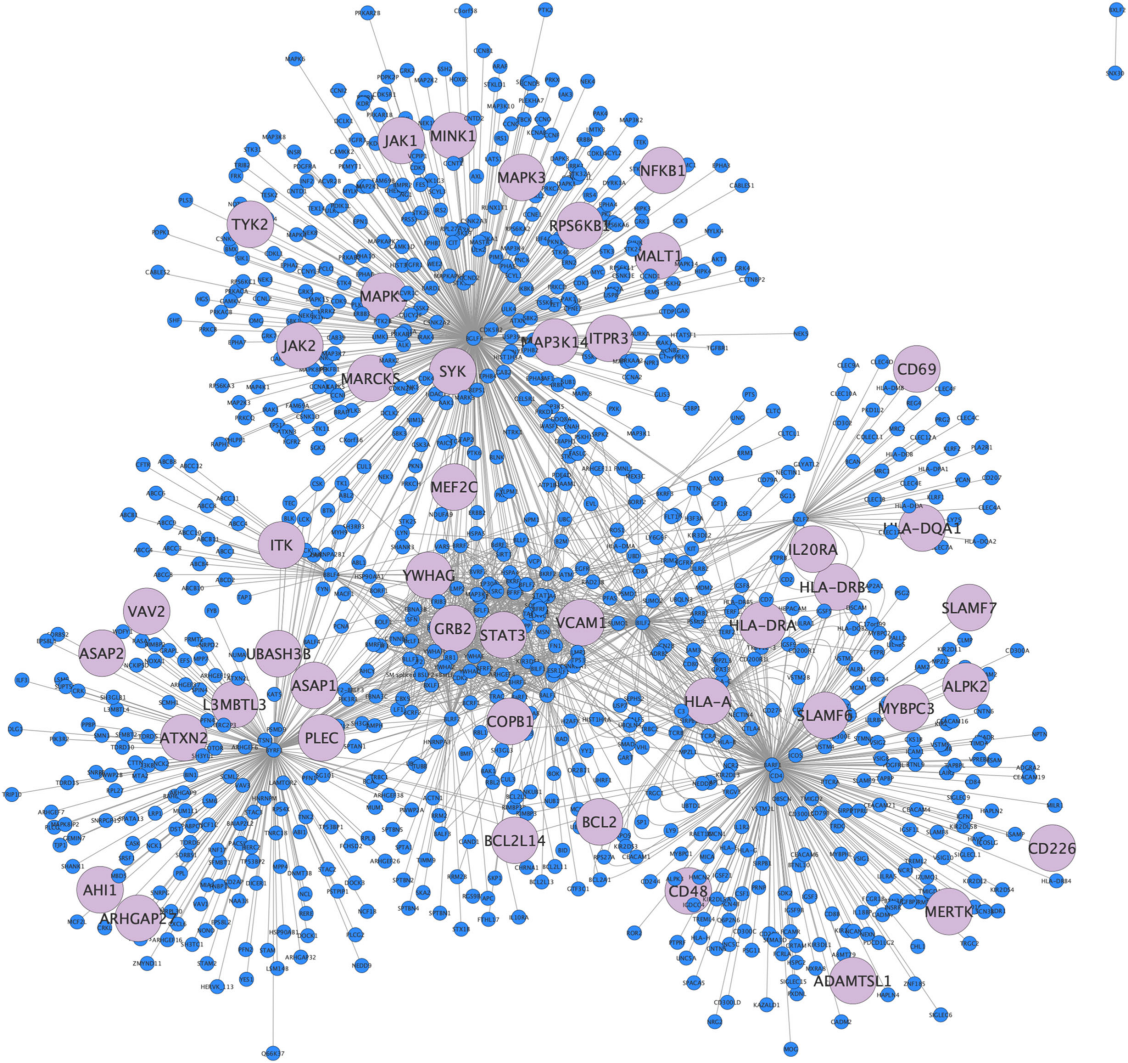
EBV interactome. EBV-Human interactome representation as a network, in light blue 45 of 958 nodes depict overlap genes from GWAS.

We observed significant overlap between EBV interactome and MS associated genes. Specifically, we found 45 genes significantly associated with MS [Odds ratio (OR)=2.2; p < 0.001]. We mapped these genes within the EBV-human interactome (**Figure 2**).

We then analyzed the genes of the interactome through “betweenness centrality”, a node centrality index that indicates the relevance of a protein according to its capacity to interact and functionally communicate with other proteins (see **Supplementary Material 1**).

Among the EBV-human interactome genes, we identified 10 out of 45 genes associated with high betweenness.

For CMV, we identified 50 genes that exhibited a significant association with MS (OR=2.0; p < 0.001). While both EBV and CMV associations with MS genes proved to be statistically significant, EBV yielded a higher degree of association (and therefore, a higher statistical reliability) (**Supplementary Material 2**, **Supplementary Figure 1**).

Adenovirus 7 (which served as a negative control) shared only 5 genes in common with GWAS, with no statistically significant association (OR=1.1; p = 0.5) (**Supplementary Material 2**).

### 3.2 Glial and immune cells gene expression and pathway analysis

**Figure 3:**
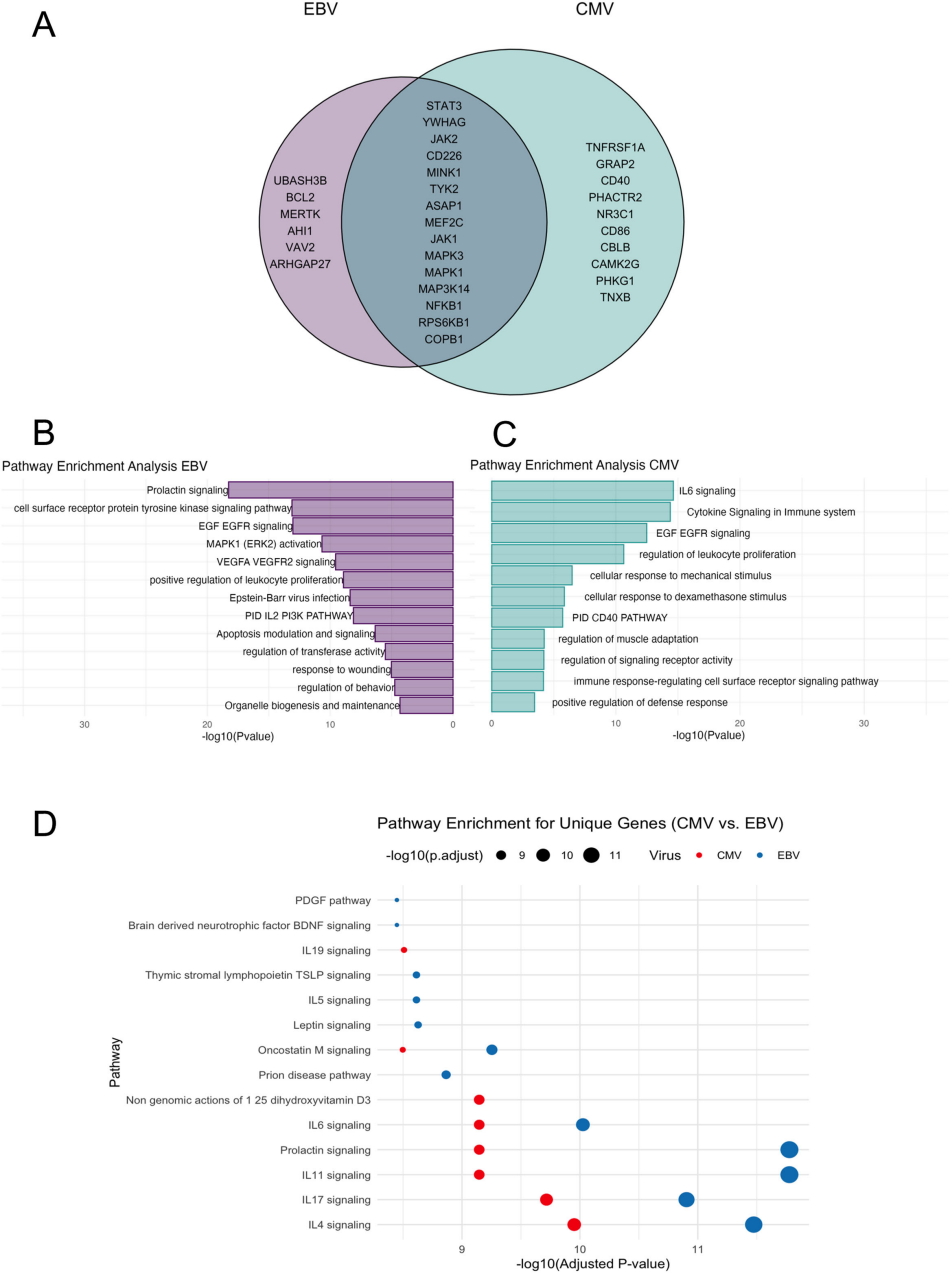
Gene pathway analysis. **(A)** Venn-diagram: in the pink circle only genes of EBV, in the green circle only genes of CMV. The intersection represents genes shared by the two viruses. **(B)** Representation of the most significant pathways for genes overlapping with EBV and associated with MS, bar graph of enriched terms across input gene lists for EBV, it shows the -log10 (p-value) on the x-axis, indicating the significance of each pathway. **(C)** Bar graph of enriched terms across input gene lists for CMV -log10 (p-value) on the x-axis. **(D)** Bubble Plot shows pathway enrichment for unique genes of CMV vs. EBV. The x-axis represents -log10 (adjusted p-values), while the size of the bubbles corresponds to the magnitude of -log10 (adjusted p-values). The color differentiates between the two viruses: in red circles the CMV and in blue circles the EBV.

Among the various immune and glial cell populations, we identified a “core” set of 21 genes that represent the intersection between the EBV interactome and MS-associated genes, which are expressed across all the selected cell types. For CMV, we identified a set of 25 genes expressed in all the analyzed immune and glial cells. Notably, 15 genes were found to be shared between the two viruses (**Figure 3A**).

Subsequently, we conducted a functional enrichment analysis for EBV associated genes using Metascape, a web-based utility that employs the Molecular Complex Detection (MCODE) algorithm. This approach allows for module analysis, contributing to the identification of dense regions within protein interaction networks. The overrepresented pathways (**Figure 3B**) are the prolactin pathway, cell surface receptor protein tyrosine kinase signaling pathway, EGF EGFR signaling, MAPK1 (ERK2) activation, VEGFA, VEGFR2 signaling, positive regulation of leukocyte proliferation, and EBV infection (**Supplementary Material 3**). Among CMV-MS interactome. We found, as most represented pathways, the IL-6 signaling pathway, cytokine signaling in the immune system, EGF-EGFR signaling, and regulation of leukocyte proliferation (**Figures 3C, D**) (**Supplementary Material 4**).

We then analyzed the top enriched pathways for CMV and EBV, showing the unique pathways for each virus based on their respective pathway enrichment results from Reactome, Gene Ontology (GO), and WikiPathways. EBV- and CMV-related genes were involved in largely the same pathways, but with varying degrees of significance. This analysis also revealed pathways uniquely related to EBV or CMV, respectively. Notably, CMV-related pathways are related to non-genomic actions of 1-25-dihydroxyvitamin D3 and IL-19 signaling, while EBV-related genes are involved in neurodegenerative and neurotrophic pathways, such as as prion disease pathway, leptin pathway, brain derived neurotrophic factor (BDNF) signaling, and pathways for maintenance of survival and functions of B cell, T cells and platelets, such as the IL-5 signaling pathway, thymic stromal lymphopoietin TSLP signaling, and PDGF signaling.

**Figure 4:**
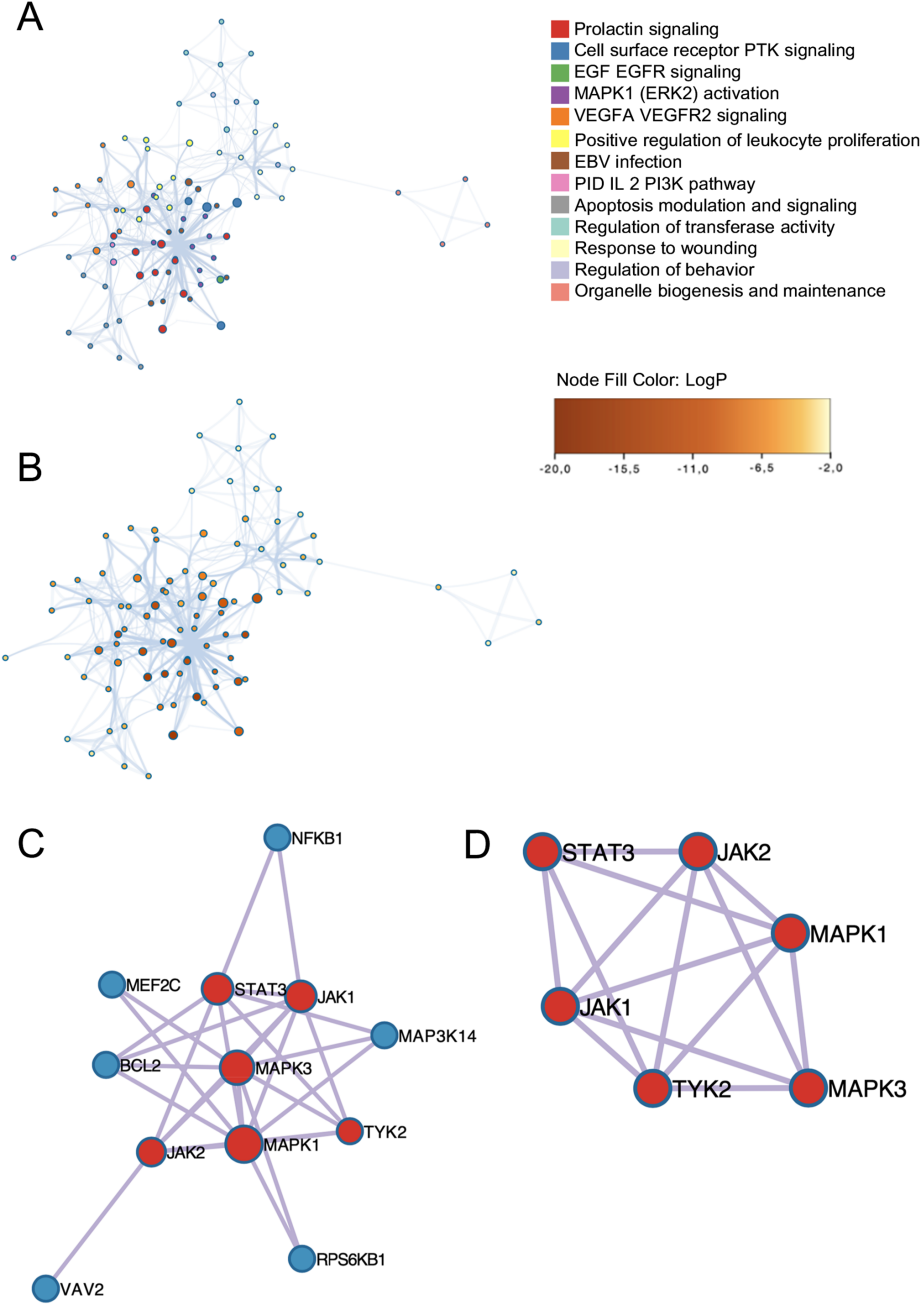
Subset cluster network representation. The size of each node is proportional to the number of input genes associated with that term and **(A)** EBV genes network of enriched terms colored by cluster ID, the color indicates the cluster to which each node belongs. Where nodes that share the same cluster ID are typically close to each other. **(B)** EBV enrichment network nodes colored by p-value instead, depicts the degree of significance of the enrichment network, as sorted by node. Visualization of PPI network analysis using the MCODE algorithm. Each node represents a protein, while edges indicate interactions. The identified clusters suggest potential protein complexes or functional groups that may play significant roles in the biological processes. **(C)** EBV Protein-protein interaction network. **(D)** EBV MCODE components identified in the gene lists colored by cluster.

The top representative terms from WikiPathways, GO, and Reactome were selected from the clusters and visualized as a network for both EBV and CMV. For EBV, the most significant associated pathways were the prolactin signaling pathway, the receptor tyrosine kinase, the EGFR signaling pathway (a signal transduction pathway that plays a crucial role in the regulation of cell growth, proliferation and survival), and the MAPK1 activation one, which is involved in regulating various cellular processes, including response to external stimuli (**Figure 4**). On the other hand, the most important pathways associated with CMV included the IL-6 signaling pathway, the cytokine signaling pathway in the immune system and, similarly to EBV, the EGF-EGFR signaling pathway (**Figure 4**).

**Figure 5:**
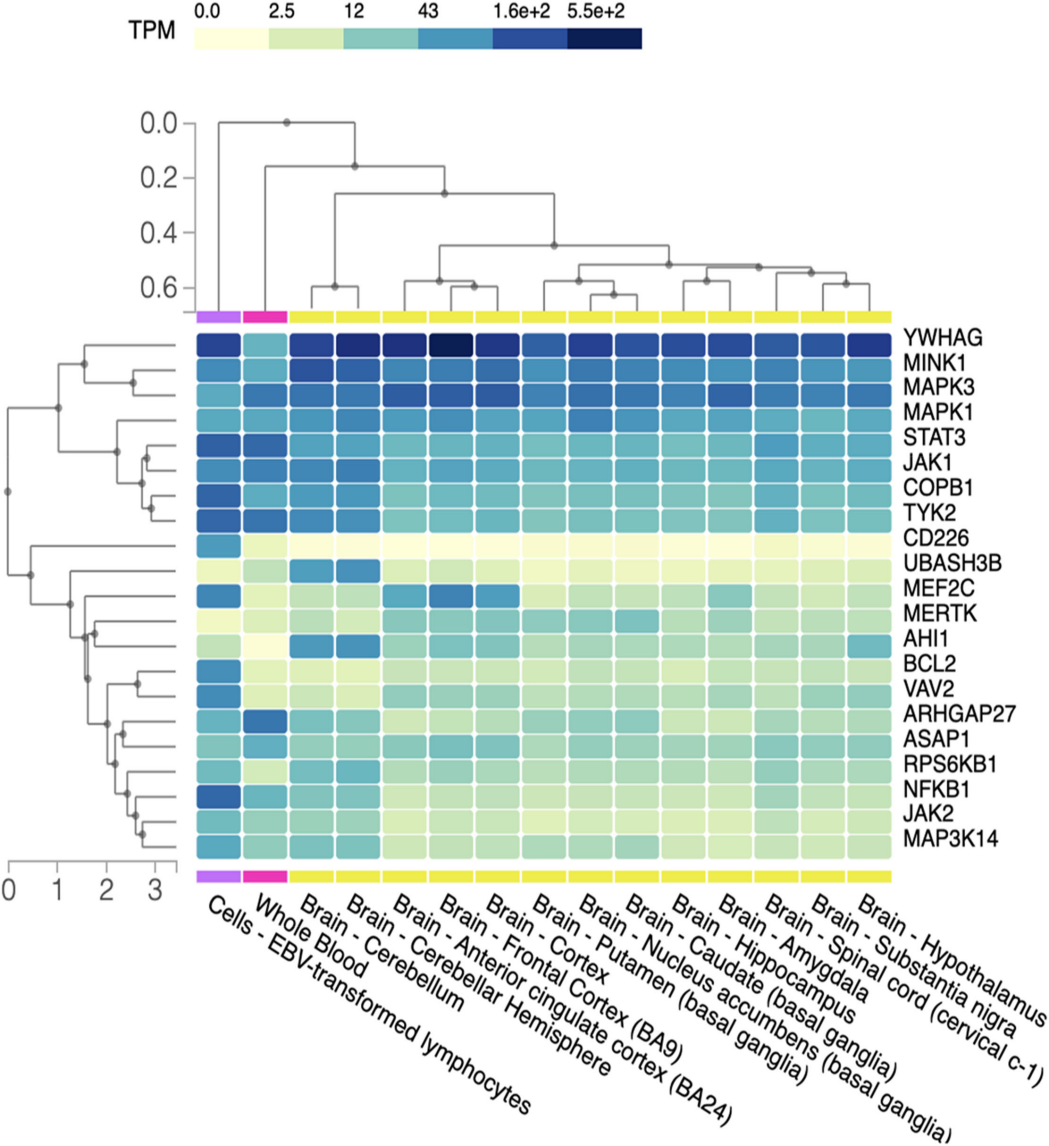
Gene expression in CNS. Heat map representation of gene expression level in different brain regions, blood cells (negative control) and lymphocytes EBV-infected (positive control). Different regions of the CNS are listed on the x-axis while on the y-axis we have the group of genes. TPM (transcripts per million) gives an idea of how much the genes are transcribed in the different regions of CNS, visually represented with an increasing color gradation from yellow to dark blue.

For both EBV and CMV, M-CODE algorithm was then applied to this network to identify neighborhoods where proteins are densely connected. All protein-protein interactions among input genes were extracted from PPI data source and formed a PPI network. GO enrichment analysis was applied to the network to extract biological meanings. The resultant network comprises a subset of proteins involved in physical interactions with at least one other member in the list (**Figure 5**).

### 3.3 Tissue gene expression in CNS

Through GTEx, we obtained a heatmap representing various degrees of gene expression in respect to the different regions of the CNS (**Figure 5**).

We observed that, compared to the positive control (EBV-infected lymphocyte), CD226, BCL2, VAV2, RPSKB1, JAK2, MAPK3K14 and NFKB1 exhibit widespread downregulation in various regions of the CNS. Conversely, YWHAG is extensively expressed alongside MINK1 and, to a lesser extent, MAPK3 and MAPK1. Some genes display selective expression in specific areas: UBASH3B, that encodes a protein that inhibits endocytosis of the epidermal growth factor receptor and has been linked to immune driven diseases such as Behçet’s and systemic lupus erythematosus (35) is overexpressed in the cerebellum hemisphere, while AHI1, that is involved in vesicle trafficking and appears to be essential for both cerebellar and cortical development (36), shows upregulation not only in the cerebellum, but also in the hypothalamus, albeit at lower levels. Finally, the MEF2C gene, encoding for one of the transcription factors of the MADS-BOX family involved in brain development and neuronal formation and differentiation (37), is underexpressed across all the analyzed areas, except for the anterior cingulate cortex and the frontal cortex.

### 3.4 MS transcriptomes analysis

Finally, we retrieved the expression of EBV-related genes in selected CNS and blood transcriptomic studies performed on samples from MS patients and controls from GEO.

We found a statistically significant degree of upregulation (p < 0.05) for MINK1 (a serine/threonine kinase) (misshapen like kinase 1) and ASAP1 (ArfGAP with SH3 domain, ankyrin repeat and PH domain 1) in microglial cells. In astrocytes, BCL2 (B-cell lymphoma 2) was upregulated, while AHI1 (Abelson helper integration site 1) was downregulated in MS vs controls. For oligodendrocytes, we did not find any significant degree of up or downregulated genes belonging to the EBV interactome. For peripheral blood cells, we found upregulation of ARHGAP27 (Rho GTPase-activating protein 27) in CD4+ and CD8+ lymphocytes in MS patients compared to negative controls (idiopathic intracranial hypertension patients).

## 4 Discussion

While the role of EBV as a precondition for the development of MS has been established (3), the link between the virus and the neuroinflammatory mechanisms driving the disease remains elusive.

Several studies have investigated the genetic and molecular interactions between EBV and MS, but genomic and transcriptomic approaches remain limited. Notably, Huang et al. used a genomic approach by analyzing GWAS and HLA alleles, highlighting the role of HLA-DRB1*15:01 in EBV immunity and MS susceptibility (38). Transcriptomic studies have provided further insight into EBV’s role in MS. Afrasiabi et al. demonstrated that EBV infection alters the expression of multiple MS risk loci, with EBNA2 directly regulating certain risk SNPs (39). Similarly, Harley et al. found that EBNA2 and its associated transcription factors occupy several autoimmune risk loci, reinforcing the idea that EBV-infected B cells contribute to immune dysregulation in MS (40). Together, these studies highlight the interplay between genomic susceptibility and transcriptomic regulation in EBV-driven MS pathogenesis. Building on these previous findings, our study takes a novel approach by applying a network-based analysis to investigate the interactome involved in EBV and MS pathogenesis. To the best of our knowledge, this is the first study to use this method, allowing us to identify genes and pathways linked to the EBV-MS association that have not been thoroughly explored in the literature so far.

In the present study, we used P-HIPSTer as a data source, due to the greater inclusivity. This database provides a user-friendly interface to run the search by virus and has the characteristic of relying on predicted PPI (prePPI) rather than PPIs, as in other databases. This feature enhances its potential for uncovering a broader range of interactions. Overall, inclusivity was deemed advantageous to mitigate the risk of overlooking potentially significant interactions. Furthermore, prePPI is a unique resource that generates novel hypotheses for the existence of PPIs, both direct and indirect, and it has already proved to be a reliable source of novel interactions, with a precision of prediction of almost 75–80% (20, 41).

The analysis of the intersection between the EBV interactome and GWAS-identified genes revealed several MS-associated genes that appeared to be central to the network. Notably, the HLA-DRB1 gene, which is well known for its association with MS susceptibility (37), exhibited high betweenness within the interactome.

Through our analysis of biological pathways, we encountered both predictable and novel results. Among the various pathways associated with EBV-related genes, we identified several pathways involved in immune system regulation, including the tyrosine kinase activation pathway. This is particularly noteworthy given the ongoing research on Tyk inhibitor drugs in MS (42).

In our study, we identified the prolactin (PRL) signaling pathway as being associated with both EBV and, to a lesser extent, CMV. This pathway is particularly interesting due to its involvement in the increased prevalence of immune-mediated diseases in females, which are strongly influenced by hormonal status (43) and plays a significant role in disease progression (44, 45).

The differences in the prevalence and severity of MS between males and females are driven by complex and poorly understood interactions between genetic, hormonal, and environmental factors. Some insights into this have been provided by the study of Català-Senent et al., which aimed to identify sex-related molecular mechanisms underlying MS pathogenesis. This was achieved through the identification of transcriptomic and functional differences between male and female MS patients (46). The role of hormones in the pathophysiology and course of MS is further highlighted by clinical observations in female MS patients during pregnancy. Notably, the risk of relapse significantly declines during the third trimester but increases threefold in the first 3–4 months after delivery. During pregnancy, hormonal levels fluctuate considerably; for instance PRL levels rise during pregnancy and reach peak values after delivery. Typically, PRL and estrogens act as immune stimulants, while progesterone and testosterone exert a suppressive role (47). Prolactin is synthesized in an extra-pituitary source by other tissues such as the decidua, the adipose tissue, skin follicles, immune cells, and the brain, e.g. in the hypothalamus and cerebellum (48). In the experimental autoimmune encephalomyelitis (EAE), a widely used MS animal model, plasma prolactin levels correlate with disease severity (49). Other findings reported a reactivation of EBV in response to stress hormones (as PRL) increase, through a switch from latency to replication by immunomodulatory mechanisms (50, 51). Furthermore, lymphocyte proliferation and macrophage activation may be reduced by either antibodies against prolactin or suppression of the release of prolactin from the pituitary gland, thus suggesting that prolactin can lead to a rise in lymphocyte counts counteracting the immunosuppressive action of elevated cortisol levels (52).

In the enrichment analysis of CMV-MS genes, we found some biological pathways closely associated with immune system activity and regulation such as the IL-6 and CD40 signaling pathway. IL-6 stimulates Th17 differentiation and inhibits Tregs generation. In MS lesions, IL-6 is produced not only by CNS infiltrating leukocytes, but also locally by microglia and astrocytes (53). On the other hand, CD40 encodes for a surface receptor involved in the immune response, particularly in the activation of B lymphocytes (54), and regulates adaptive and innate immune responses. Of note, CD40 is currently a promising target of novel emerging MS treatments such as frexalimab (55).

We also identified biological pathways uniquely associated with only one of the two viruses, such as the vitamin D pathway linked to CMV. Vitamin D plays a crucial role in regulating immune function, and low levels are associated with an increased risk of autoimmune diseases, including MS (56). Additionally, low vitamin D levels are connected to a higher susceptibility to viral infections, including the reactivation of latent viruses like CMV and EBV. Notably, Mowry et al. (2011) recently found that sufficient vitamin D levels were associated with increased CMV antibody levels in MS/CIS patients compared to controls (57). Although the potential protective role of CMV in the development of MS is still debated, CMV infection has been linked to potential protective effects in MS, possibly due to the modulation of immune cells such as regulatory T-cells and anti-inflammatory cytokines (58–60). It is of great interest how these different risk and potentially protective factors may interact, that is, vitamin D deficiency and CMV, and it would be worthwhile to investigate how this interplay may contribute to MS susceptibility or protection from a cell biology perspective. We may suggest that CMV could not have a protective role in concomitance with low vitamin D levels, as it could contribute to an imbalanced immune response.

Next, we retrieved the EBV-interactome selected genes in transcriptomics datasets confronting MS and controls. We identified ARHGAP27 as significantly upregulated in the peripheral blood CD4+ cells of MS patients. This gene encodes for a Rho GTPase inhibitor potentially involved in regulating the cytoskeleton and cell movement (61). For microglia we analyzed transcriptomics data derived from white matter sample tissue of MS patients compared to controls. We found overexpression of ASAP1, a gene encoding for a protein closely associated with cytoskeletal reorganization, cell adhesion, and migration (62), and MINK1, which encodes for a serine/threonine kinase.

MINK1 contributes to regulate the cell cycle, apoptosis, cytoskeleton organization, cell migration, embryogenesis and tissue homeostasis; it also plays an important role in immunological responses, inhibiting Th17 and Th1 cell differentiation and regulating NLRP3 inflammasome function (63). In EAE model, MINK1^−/−^ mice showed a 2–3 times increased number of Th17 and Th1 cells compared to wild type mice; of note, microglial cells are implicated in the differentiation of Th17, that have a well-known role in EAE pathogenesis, through the production of several cytokines, including IL-6. MINK1^−/−^ mice also displayed enhanced EAE severity with more prominent demyelination in the central nervous system and the spinal cord (64). Interestingly, in the CNS, MINK1 has been identified as a component of the postsynaptic density; it regulates synaptic physiology and seems to play a role in neurodegeneration (65).

Reactive oxygen species (ROS), which can be produced by cells involved in the host-defense responses, are essential signaling molecules and inflammation mediators but are also known for promoting tissue injury and taking part in various immunological disorders (66). ROS seems to boost the expression of MINK1. The role of MINK in numerous inflammatory diseases, such as rheumatoid arthritis, asthma, SLE has been evaluated extensively (63); to investigate its role in MS could yield interesting results.

For astrocytes, we identified two genes: AHI1 and BCL2, which resulted down and upregulated in MS vs controls, respectively. AHI1 is crucial for brain development: its mutation leads to Joubert syndrome, characterized by cerebellar and brainstem malformations, as well as symptoms like breathing irregularities, hypotonia, developmental delays, and ocular motor apraxia. In addition, AHI1 has been extensively studied in depression models, where its loss-of-function mutations are linked to depressive behaviors in mice (67). In these models, the mitochondrial Ahi1/GR complex and TFAM regulate mtDNA copy numbers and brain ATP levels, and the increase of Ahi1/GR complex alleviates depressive behaviors. Besides depression models, similar mitochondrial defects were also found in MS (68, 69). Astrocyte-mediated ATP release and purinergic signaling are fundamental for communication between astrocytes, neurons, and autoreactive immune cells, particularly in EAE (70). The link between AHI1, immune function and MS is particularly interesting considering studies on major depressive disorder (MDD), characterized by an atypical immune response to EBV infection. Although these findings have primarily emerged within the context of MDD, they may also be relevant to MS due to shared mechanisms of immune dysfunction (71). For example, a reduction of AHI1 expression in macrophages from MDD patients weakens antiviral responses, as it may happen in MS. Depression-related arginine vasopressin (AVP) reduces AHI1 expression, which disrupts Tyk2 and interferon signaling, further compromising immune defense (72, 73). Interestingly, enhancing antiviral immunity with meptazinol in depression models opens new perspectives in modulating immune responses in MS. Lastly, the rs4896153T allele, which is linked to MS, reduces AHI1 RNA expression and correlates with increased IFNγ-producing CD4+ T cells (74).

We detected upregulation of BCL2 in astrocytes of MS patients compared to controls. This gene encodes for an integral outer mitochondrial membrane protein that blocks apoptosis; its overexpression is associated with lymphomas (75) and has also been found upregulated in other conditions characterized by glial activation (76). We may hypothesize that the upregulation of BCL2 in astrocytes during MS may be closely associated to reactive gliosis, that could synergically act in CNS inflammation and demyelination, e.g. in glial scar formation by hypertrophic astrocytes (77).

Some of the genes identified in our analysis have been previously noted in the literature for their potential as therapeutic targets in MS, particularly CD226 and the JAK/STAT pathway. CD226, typically overexpressed on activated CD4+ cells and crucial for Th1 differentiation (78), when targeted by anti-CD226 monoclonal antibodies, delays EAE onset (79). Additionally, reduced CD226 and T-bet expression in CD4+ cells during EAE has been linked to less CNS damage and fewer infiltrating T cells (80). Moreover, JAK2 deletion, and the resulting inactivation of STAT5, fully prevents EAE onset in mice (81). Similarly, Xue et al. demonstrated that the JAK2/STAT3 pathway blocker AG490 reduces EAE severity and peripheral inflammation (82). These findings would be worthwhile future insights for their potential application in patients.

Despite the interesting findings of our work, we acknowledge that the study has some limitations. First, the gene association repository has grown significantly in recent years, and we had to choose which resources to use based on their reliability, robustness, and comprehensiveness, such as the HPA. While using publicly available data may have excluded potentially significant unpublished datasets, we emphasize that the HPA is an appropriate choice, offering broad tissue coverage and high-quality data, which enable robust and reproducible results. Integrating additional resources may be considered for future studies to strengthen the results and enhance the conclusions. Finally, our study is primarily bioinformatics-based. Our goal was to develop a computational method that establishes a stable and reproducible pipeline for identifying new pathways and genes potentially involved in the etiopathogenesis of MS. We believe this study serves as a starting point and lays the groundwork for future experimental validation of our findings.

## 5 Conclusion

Overall, this study offers new insights into the molecular dynamics driving MS pathogenesis, with a focus on the role of EBV dysregulating gene expression in the context of abnormal immune responses and cellular function. We identified several potentially disease-associated genes that interact with EBV, particularly in astrocytes and microglia, including MINK1, BCL2, AHI1. Additionally, we uncovered gene pathways, such as the prolactin one, that may play a role in MS pathogenesis, warranting further experimental validation. The potential interactions between viruses like EBV and CMV with immune and glial cells, as explored in this study, could provide valuable new insights into the etiopathogenesis of MS. These findings may have broader implications beyond MS, as EBV has been linked to other autoimmune diseases, including systemic lupus erythematosus, rheumatoid arthritis, juvenile idiopathic arthritis, inflammatory bowel disease, celiac disease, and type 1 diabetes.

## Data Availability

The datasets presented in this study can be found in online repositories. The names of the repository/repositories and accession number(s) can be found in the article/**Supplementary Material**.
