# Supplementary figures and images for: Exploring the role of EBV in multiple sclerosis pathogenesis through EBV interactome
Source: Front Immunol. 2025 Apr 2;16:1557483. doi: 10.3389/fimmu.2025.1557483 (PMC11999961; doi:10.3389/fimmu.2025.1557483)

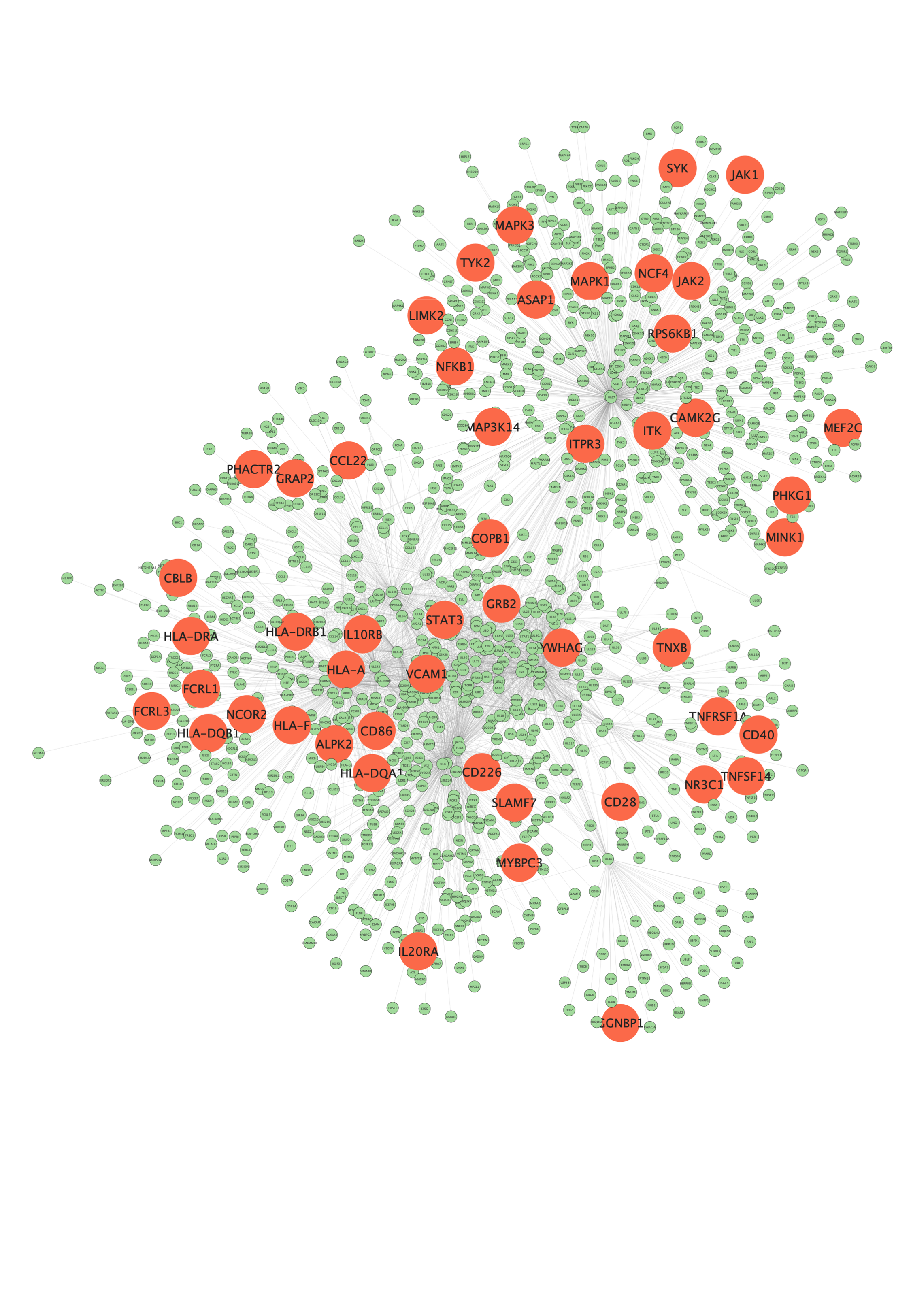

Supplement: Supplementary file 1 [file Image1.tif]
