# Supplementary material for: Exploring the role of EBV in multiple sclerosis pathogenesis through EBV interactome
Source: Front Immunol. 2025 Apr 2;16:1557483. doi: 10.3389/fimmu.2025.1557483 (PMC11999961; doi:10.3389/fimmu.2025.1557483)
Supplement: Supplementary file 3 [file Table2.docx]

**Supplementary file 2**

**Adenovirus 7**

Detailed information about this GeneOverlap object:

listA size=226, e.g. ELAVL1 YWHAQ YWHAE

listB size=577, e.g. HLA-DRB1 TNXB HLA-DQA1

Intersection size=6, e.g. YWHAG GRB2 STAT3

Union size=797, e.g. ELAVL1 YWHAQ YWHAE

Genome size=23000

# Contingency Table:

notA inA

notB 22203 220

inB 571 6

Overlapping p-value=0.5

Odds ratio=1.1

Overlap tested using Fisher's exact test (alternative=greater)

Jaccard Index=0.0

**CMV**

Detailed information about this GeneOverlap object:

listA size=1058, e.g. CX3CL1 TNFRSF10B B2M

listB size=577, e.g. HLA-DRB1 TNXB HLA-DQA1

Intersection size=50, e.g. HLA-A HLA-DRA STAT3

Union size=1585, e.g. CX3CL1 TNFRSF10B B2M

Genome size=23000

# Contingency Table:

notA inA

notB 21415 1008

inB 527 50

Overlapping p-value=1.4e-05

Odds ratio=2.0

Overlap tested using Fisher's exact test (alternative=greater)

Jaccard Index=0.0

**EBV**

Detailed information about this GeneOverlap object:

listA size=909, e.g. ZMYND11 HLA-DRA CSF1

listB size=577, e.g. HLA-DRB1 TNXB HLA-DQA1

Intersection size=46, e.g. HLA-DRA STAT3 GRB2

Union size=1440, e.g. ZMYND11 HLA-DRA CSF1

Genome size=23000

# Contingency Table:

notA inA

notB 21560 863

inB 531 46

Overlapping p-value=5.7e-06

Odds ratio=2.2

Overlap tested using Fisher's exact test (alternative=greater)

Jaccard Index=0.0
